# Prospective analysis of the expression status of FGFR2 and HER2 in colorectal and gastric cancer populations: DS-Screen Study

**DOI:** 10.1007/s00384-022-04162-2

**Published:** 2022-05-19

**Authors:** Hisateru Yasui, Atsushi Takeno, Hiroki Hara, Hiroshi Imamura, Hiroki Akamatsu, Kazumasa Fujitani, Minoru Nakane, Chihiro Nakayama Kondoh, Seigo Yukisawa, Junichiro Nasu, Yoshinori Miyata, Akitaka Makiyama, Hiroyasu Ishida, Norimasa Yoshida, Eiji Matsumura, Masato Ishigami, Masahiro Sugihara, Atsushi Ochiai, Toshihiko Doi

**Affiliations:** 1 Kobe City Medical Center General Hospital, Hyogo, Japan; 2 Kansai Rosai Hospital, Hyogo, Japan; 3 Saitama Cancer Center, Saitama, Japan; 4 Toyonaka Municipal Hospital, Osaka, Japan; 5 Osaka Police Hospital, Osaka, Japan; 6 Osaka General Medical Center, Osaka, Japan; 7 Japanese Red Cross Musashino Hospital, Tokyo, Japan; 8 Toranomon Hospital, Tokyo, Japan; 9 Tochigi Cancer Center, Tochigi, Japan; 10 Okayama Saiseikai General Hospital, Okayama, Japan; 11 Saku Central Hospital Advanced Care Center, Nagano, Japan; 12 Japan Community Healthcare Organization Kyushu Hospital, Fukuoka, Japan; 13 Gifu University Hospital, Gifu, Japan; 14 Mito Medical Center, Ibaraki, Japan; 15 Japanese Red Cross Kyoto Daiichi Hospital, Kyoto, Japan; 16 Daiichi Sankyo Co., Ltd, Tokyo, Japan; 17 National Cancer Center Exploratory Oncology Research & Clinical Trial Center, Tokyo, Japan; 18 National Cancer Center Hospital East, Chiba, Japan

**Keywords:** Colorectal cancer, FGFR2, Gastric cancer, HER2

## Abstract

**Purpose:**

Fibroblast growth factor receptor 2 (FGFR2) and human epidermal growth factor receptor 2 (HER2) proteins are both molecular targets for cancer therapy. The objective of this study was to evaluate the expression status of FGFR2 and HER2 in patients with gastric cancer (GC) or colorectal cancer (CRC).

**Methods:**

Archived tumor tissue samples from patients with histologically-confirmed GC or CRC suitable for chemotherapy were analyzed for FGFR2 and HER2 expression using immunohistochemistry and fluorescence in situ hybridization (HER2 in CRC only).

**Results:**

A total of 176 GC patients and 389 CRC patients were enrolled. Among patients with GC, 25.6% were FGFR2-positive and 26.1% were HER2-positive. Among patients with CRC, 2.9% were FGFR2-positive and 16.2% were HER2-positive. No clear relationship was found between FGFR2 and HER2 status in either GC or CRC. In GC, FGFR2 and HER2 statuses did not differ between different primary cancer locations, whereas there were some differences between histological types. Based on FGFR2- and/or HER2-positive status, 117 patients were identified as potentially suitable for inclusion in clinical trials of therapeutic agents targeting the relevant protein (*GC* = 45, *CRC* = 72; *FGFR* = 56, *HER2* = 62), of whom 7 were eventually enrolled into such clinical trials.

**Conclusions:**

This study indicated the prevalence of FGFR2 and HER2 in GC and CRC in the Japanese population. The screening performed in this study could be useful for identifying eligible patients for future clinical trials of agents targeting these proteins.

**Trial registration:**

Clinical trial registration Japic CTI No.: JapicCTI-163380. https://www.clinicaltrials.jp/cti-user/trial/ShowDirect.jsp?directLink=RNlzx1PPCuT.PrVNPxPRwA.

**Supplementary Information:**

The online version contains supplementary material available at 10.1007/s00384-022-04162-2.

## Introduction

An increasing number of molecular-targeted therapies are available in the field of oncology. When using such treatments in the clinical setting, it is desirable to be able to identify those patients in whom the target molecule is expressed and who are therefore expected to benefit from the therapy.

Fibroblast growth factor receptor 2 (FGFR2) and human epidermal growth factor receptor 2 (HER2) proteins are well-known molecular targets for cancer therapy. FGFR2 consists of an extracellular ligand-binding region consisting of three Ig-like domains, a single transmembrane region, and an intracellular tyrosine kinase region [1]. Various cellular functions, including cell proliferation, migration, and differentiation, are regulated by the FGF signaling pathway [1, 2]. HER2, a receptor tyrosine kinase belonging to the epidermal growth factor receptor family, is involved in regulating the proliferation and differentiation of normal cells and also acts as an oncogene, driving gene amplification and mutation [3]. Novel agents targeting these proteins are emerging, including several next-generation antibodies with enhanced antibody-dependent cellular cytotoxicity (ADCC) activity [4] and antibody–drug conjugates [5, 6].

HER2 is overexpressed in 10–20% of gastric cancers (GCs), and assessment of HER2 status is necessary to identify patients eligible for treatment with drugs such as trastuzumab [7]. Less is known about the expression status of FGFR2 in GC or about HER2 and FGFR2 in other gastrointestinal cancers, such as colorectal cancer (CRC). Accurate characterization of HER2 and FGFR2 expression in specific types of cancer is important for determining the relevance of these proteins as markers for identifying potential candidates for treatment with relevant targeted therapies. Furthermore, to optimize the clinical development strategies of these emerging agents, it would be useful to investigate FGFR2 and HER2 expression patterns, such as whether they are co-expressed or expressed in a mutually-exclusive manner.

The primary objectives of this study were to investigate the expression status of FGFR2 and HER2 in tissue samples from patients with GC or CRC, and to evaluate the relationship between background factors and protein expression status. A secondary objective was to identify patients who were potentially eligible for clinical trials involving the therapeutic agents DS-1123 (a monoclonal antibody directed against FGFR2) or trastuzumab deruxtecan (DS-8201, T-DXd; an antibody–drug conjugate with a HER2 antibody, tetrapeptide-based cleavable linker, and a novel topoisomerase I inhibitor payload).

## Patients and methods

This prospective, multicenter study, which was conducted in a routine clinical practice setting between November 2016 and June 2018, enrolled patients aged ≥20 years with histologically confirmed GC (including gastroesophageal junction [GEJ] cancer) or CRC for which chemotherapy was indicated. Enrollment of GC patients was stopped on April 2017 due to completion of a relevant clinical trial, DS1123-A-J101 (NCT02690337). To be eligible, patients were required to have archived tumor tissue samples that had been collected during surgery, endoscopy, or needle biopsy, and that were preserved as formalin-fixed paraffin-embedded blocks. Patients judged by the investigator to be inappropriate as study subjects were excluded.

The study was conducted in accordance with the principals of the Declaration of Helsinki. Ethical approval was obtained from the relevant ethical review board for each participating center, and patients provided written informed consent. Japic CTI No.: JapicCTI-163380.

### Patient data

Patient details were collected using an electronic data capture system. This included demographics (age, sex); tumor characteristics (histopathological diagnosis and date of diagnosis, primary location, major histological type, stage, HER2 expression [for any patients with GC who had been tested for HER2 prior to the study], and presence/absence of *rat sarcoma viral oncogene homolog* (*RAS gene*) mutations [for any patients who had been confirmed as having a mutation prior to the study]); and tumor sampling information (date and location of sampling, primary or metastatic).

### Tumor sample analysis

FGFR2 and HER2 expressions in GC and CRC samples were assessed using immunohistochemistry (IHC) at a central laboratory (SRL Medisearch Inc., Japan). For GC, the results of HER2 IHC performed at local laboratories were also collected, where available (HER2 IHC in GC patients is performed in routine clinical practice in Japan). In addition, samples from patients with CRC enrolled before December 2017 which were found to be HER2 IHC 2 + (in ≥ 10% cells) were assessed for *HER2* gene amplification using fluorescence in situ hybridization (FISH) at a central laboratory, if the patient had a suitable sample for testing and provided consent. Samples from patients with CRC enrolled after December 2017 were all assessed for HER2 FISH, regardless of HER2 IHC score.

IHC staining for FGFR2 was performed using a mouse chimeric anti-FGFR2 antibody produced in-house with Agilent DAKO EnVision™ FLEX + Mouse (LINKER) and Agilent DAKO Autostainer Link 48, which captures FGFR2 isoforms IIIb and IIIc. IHC staining for HER2 was performed using the Ventana I-VIEW pathway HER2 (4B5) kit. IHC scoring was evaluated based on three elements — IHC staining intensity, cellularity, and location — at the National Cancer Center Exploratory Oncology Research and Clinical Trial Center, Chiba, Japan (Online Resource). FISH was performed using Abbott PathVysion® HER2 DNA probe kit. *HER2* amplification was considered positive if the ratio of *HER2*/*CEP17* was ≥ 2.0 when counting total number of *HER2* and *CEP17* signals in at least 20 tumor cells.

FGFR2 positivity was defined as FGFR2 IHC 1 + to 3 + for both GC and CRC. HER2 positivity was defined as IHC 2 + or 3 + in GC, and HER2 IHC 2 + or 3 + in ≥ 10% of cells in CRC (Online Resource).

### Clinical trial participation

Patients who were found to be potentially eligible for participation in clinical trials of therapeutic agents targeting the relevant protein (i.e., GC patients with FGFR2 IHC 1 + to 3 + and CRC patients with FGFR2 IHC 1 + to 3 + or HER2 IHC 2 + or 3 + in ≥ 10% of cells) were referred to ongoing clinical trials. Information on whether these patients went on to participate in the relevant clinical trial, including any reason for non-participation, was recorded.

### Study outcomes

The primary endpoint was the percentage of patients with tumor samples in which FGFR2/HER2 protein expression was confirmed. In addition, the relationship between patient background factors and FGFR2/HER2 expression was also evaluated. Secondary endpoints included the proportion of patients with confirmed FGFR2 and HER2 expression who subsequently participated in clinical trials of therapeutic agents targeting the relevant protein.

### Statistical analysis

FGFR2 and HER2 expression statuses were analyzed descriptively using central laboratory data. Summary statistics for patient characteristics and protein expression included mean and standard deviation, median (minimum‒maximum), and number (percentage), as appropriate.

## Results

A total of 565 patients (*GC* = 176, *CRC* = 389) were enrolled in the study between November 2016 and June 2018 from 15 sites across Japan. The full analysis set (FAS) included 560 patients (176 with GC, including 16 GEJ patients, and 384 with CRC); the majority of patients had stage III or IV GC or CRC (Table 1). Five patients were excluded from the FAS because of discontinuation after informed consent due to disease worsening or no available tissue sample to submit.

**Table 1:** Patient demographics

**Parameter**	**Gastric cancer**^**a**^** (*n* = 176)**	**Colorectal cancer (*n* = 384)**	**Total (*n* = 560)**
Age (years)			
Mean ± SD	67.4 ± 9.5	62.1 ± 10.3	63.8 ± 10.3
Median (range)	68.0 (34–93)	64.0 (29–83)	66.0 (29–93)
Female, *n* (%)	39 (22.2)	172 (44.8)	211 (37.7)
Stage, *n* (%)			
I	3 (1.7)	3 (0.8)	
II	12 (6.8)	38 (9.9)	
III	47 (26.7)	86 (22.4)	
IV	114 (64.8)	251 (65.4)	
Unknown	0 (0)	6 (1.6)	
Systemic chemotherapy prior to sampling, *n* (%)	27 (15.3)	55 (14.3)	82 (14.6)
History of radiation therapy to sampling location, *n* (%)	0 (0.0)	10 (2.6)	10 (1.8)

^a^Gastric cancer included 16 patients with gastroesophageal cancer

### Gastric cancer

Among patients with GC, 25.6% (45/176) were FGFR2-positive and 26.1% (46/176) were HER2-positive. Among the subgroup with GEJ, 43.8% (7/16) were positive for FGFR2 and 43.8% were positive for HER2.

**Fig. 1:**
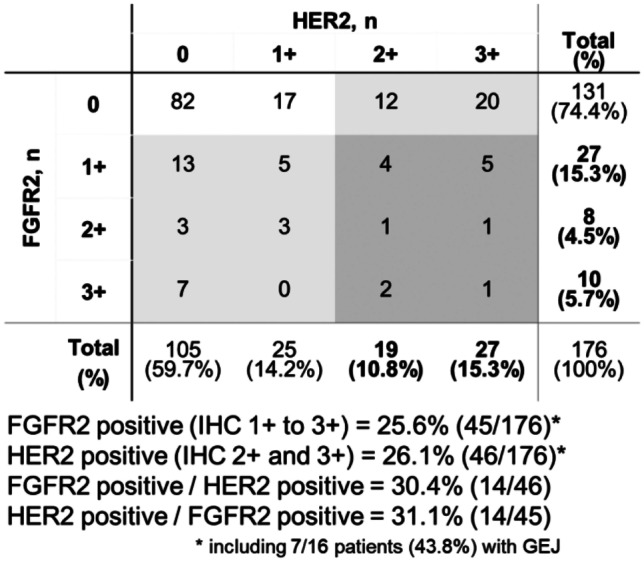
Patient distribution by FGFR2 and HER2 IHC scores in gastric cancer

There was no clear association between FGFR2 and HER2 status in patients with GC (Fig. 1). The proportion of FGFR2-positive patients among the whole population was 25.6% and among the HER2-positive population it was 30.4%. Similarly, the proportion of HER2-positive patients among the whole population was 26.1% and among the FGFR2-positive population it was 31.1%.

**Fig. 2:**
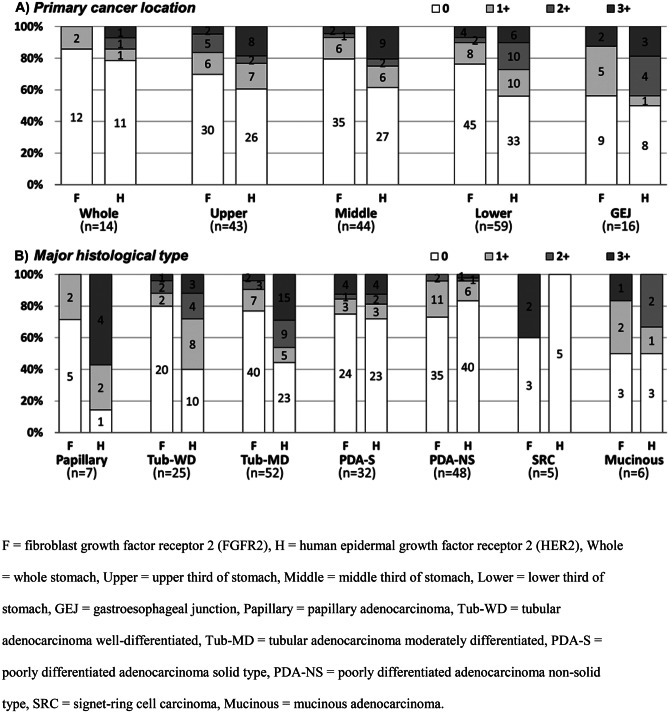
FGFR2 and HER2 IHC scores by primary location and major histology in gastric cancer

FGFR2 and HER2 statuses did not differ substantially between different primary cancer locations (Fig. 2A). With respect to major histological types, although sample numbers are low in these observations, the proportion of FGFR2-positive patients was numerically higher than that of HER2-positive patients for signet-ring carcinoma. On the other hand, for papillary adenocarcinoma and moderately differentiated tubular adenocarcinoma, the proportions of HER2-positive patients were numerically higher than those for FGFR2-positive patients (Fig. 2B).

### Colorectal cancer

Among patients with CRC, 2.9% (11/383) were FGFR2-positive and 16.2% (62/383) were HER2-positive.

**Fig. 3:**
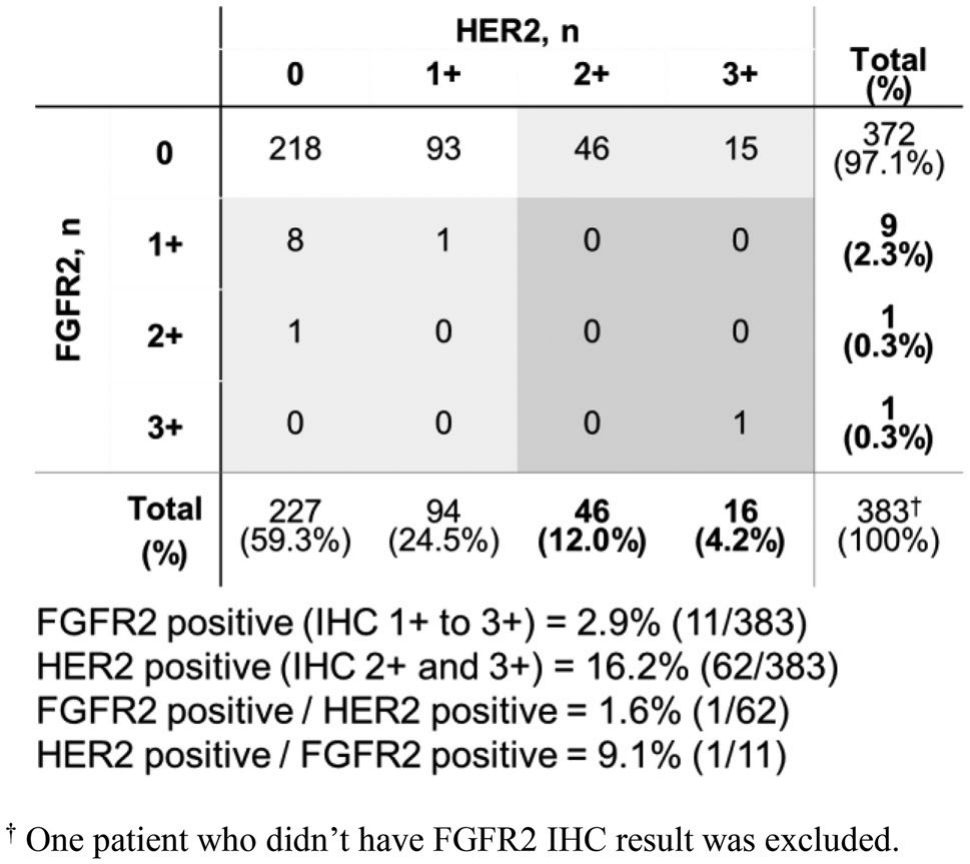
Patient distribution by FGFR2 and HER2 IHC scores in colorectal cancer

There was no clear association between FGFR2 and HER2 status in patients with CRC, although firm conclusions could not be drawn because of the small number of FGFR2-positive patients (Fig. 3). The proportion of FGFR2-positive patients among the whole population was 2.9% and among the HER2-positive population it was 1.6%. Similarly, the proportion of HER2-positive patients among the whole population was 16.2% and among the FGFR2-positive population it was 9.1%.

**Fig. 4:**
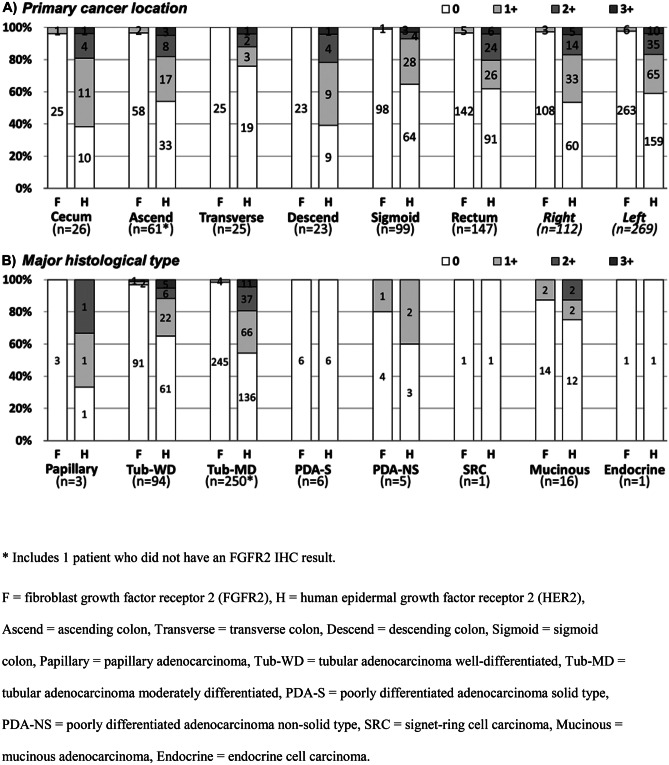
FGFR2 and HER2 IHC scores by primary location and major histology in colorectal cancer

No differences in FGFR2 or HER2 status according to primary cancer location or histological type could be identified because of the small number of FGFR2-positive patients (Fig. 4A, B). HER2 status appeared to be the same for right-side and left-side primary cancer locations (Fig. 4A).

### Referral to clinical trials

Among the study population, 117 patients were identified as being potentially suitable for inclusion in clinical trials, based on FGFR2- and/or HER2-positive status (*GC* = 45, *CRC* = 72; FGFR = 56, HER2 = 62 [1 patient was FGFR2- and HER2-positive]). Ultimately, 7 of these patients (*GC* = 4, *CRC* = 3) were enrolled into clinical trials (Table 2).

**Table 2:** Determination of eligibility and enrolment of FGFR2- or HER2-positive patients into clinical trials

**Parameter, *n* (%)**	**Gastric cancer (*n* = 176)**	**Colorectal cancer (*n* = 384)**	**Total (*n* = 560)**
FGFR2 IHC 1 + to 3 +	45 (25.6)	11 (2.9)	56 (10.0)
HER2 IHC 2 + or 3 + in GC, or HER2 IHC 2 + or 3 + in ≥ 10% of cells in CRC	46 (26.1)	62 (16.1)	108 (19.3)
Potentially suitable for clinical trials^a^	45 (25.6)	72 (18.8)	117 (20.9)
Referral to clinical trials^b^	5 (2.8)	4 (1.0)	9 (1.6)
Enrolled into clinical trials	4 (2.3)	3 (0.8)	7 (1.3)

*CRC* colorectal cancer, *FGFR2* fibroblast growth factor receptor 2, *GC* gastric cancer, *HER2* human epidermal growth factor receptor 2, *IHC* immunohistochemistry

^a^For gastric cancer, only FGFR2-positive patients were potentially eligible

^b^The major reasons that patients were not referred to clinical trials were as follows in descending order: the clinical trial recruitment period had ended, patients had been continuing prior therapy at the time of entry, or primary disease had worsened

### Other findings

**Fig. 5:**
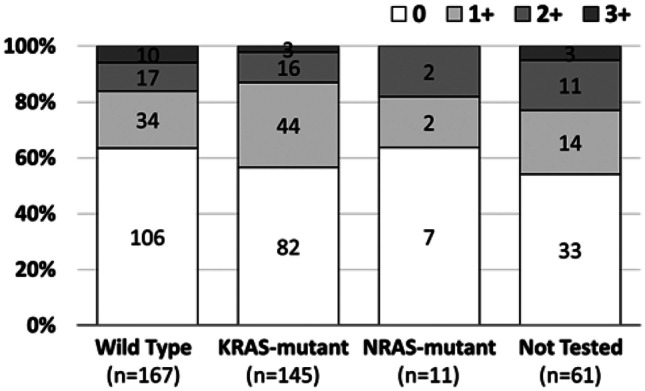
HER2 IHC scores by *RAS* mutation status in colorectal cancer

Among patients with CRC, the proportion with HER2 IHC 3 + tumors was higher in those who were *KRAS*/*NRAS* wild type positive (10/167) compared to those who were positive for *KRAS* or *NRAS* mutants (3/156), although the prevalence of HER2 IHC 3 + in these populations was low (Fig. 5).

Among patients with CRC, all those who were HER2 IHC 3 + were also HER2 amplified using FISH (*n* = 3). There were also some patients who were HER2 amplified using FISH in patients who were HER2 IHC 0, 1 + , and 2 + (Table 3).

**Table 3:** HER2 IHC scores and FISH status in colorectal cancer

	**HER2 IHC scores, *n* (%)**			
	**0 (*n* = 44)**	**1 + (*n* = 21)**	**2 + (*n* = 15)**	**3 + (*n* = 3)**
HER2 FISH status^a^				
Positive^b^	1 (2.3)	2 (9.5)	1 (6.6)	3 (100)
Negative	43 (97.7)	19 (90.5)	14 (93.3)	0 (0)

*FISH* fluorescence in situ hybridization, *HER2* human epidermal growth factor receptor 2, *IHC* immunohistochemistry

^a^HER2 FISH analysis was done for 83 CRC patients

^b^In the FISH analysis, HER2/CEP17 ratio ≥ 2 was defined as positive

**Fig. 6:**
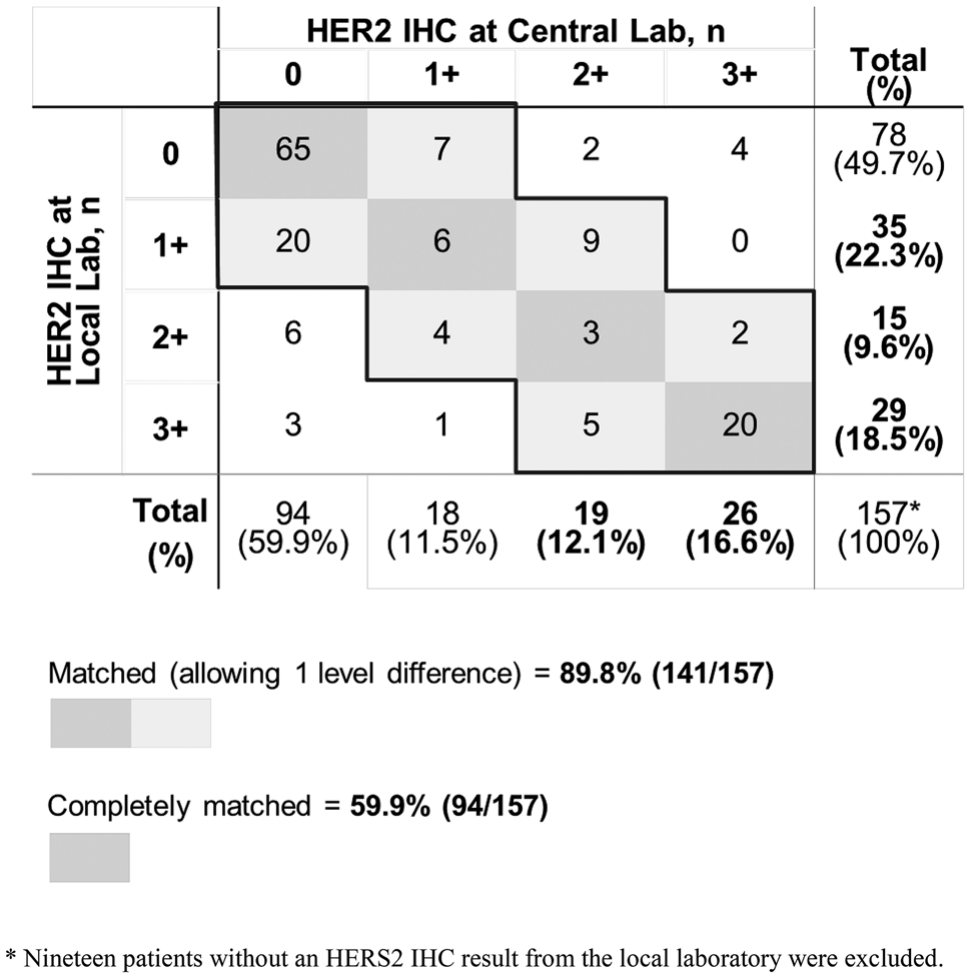
HER2 IHC scores from local and central laboratories for gastric cancer

In an assessment of the concordance of IHC scores for GC samples tested at local and central laboratories, the rate of matched HER2 IHC scores (allowing 1 level difference) was 89.8% and the rate for completely matched cases was 59.9% (Fig. 6). The rate of concordance was not affected by a difference in the sample collection date between the laboratories or by the type of IHC testing kit used (Table 4).

**Table 4:** IHC scores concordance between local and central testing in gastric cancer

	**Concordance between IHC scores from local and central testing,** ***n* (%)**	
	**Matched **^**a**^	**Completely matched**
Difference between sample collection dates, days		
0 (*n* = 107)	94 (87.9)	63 (58.9)
>0^b^ (*n* = 50)	47 (94.0)	31 (62.0)
IHC kit for local laboratory testing^c^		
Ventana I-VIEW pathway HER2 (*n* = 98)	90 (91.8)	55 (56.1)
Daco HercepTest II (*n* = 32)	30 (93.8)	20 (62.5)
Histofine HER2 kit (poly) (*n* = 4)	4 (100.0)	3 (75.0)
Unknown (*n* = 6)	6 (100.0)	5 (83.3)
Others (*n* = 17)	11 (64.7)	11 (64.7)

*HER2* human epidermal growth factor receptor 2, *IHC* immunohistochemistry

^a^ “Matched” allows 1 level difference

^b^Mean 196.7 ± 258.7 days; median 51.5 days (min 1, max 842 days)

^c^Central laboratory testing used Ventana I-VIEW pathway HER2

## Discussion

There is a need for additional information about the expression status of FGFR2 and HER2 in patients with gastrointestinal cancers. In our study, we evaluated patients with GC and CRC being managed in routine clinical practice. This is the first study to concurrently investigate the protein expression status of FGFR2 and HER2 in human tumor tissue from patients with CRC.

The HER2 positivity rate of 26.1% in GC found in the study is consistent with previous reports of HER2 overexpression of 10–23% [7–9]. HER2 expression is reported to be more common in GEJ cancer than in cancer located within the stomach [8, 9], and the rate in the GEJ subgroup in our study is consistent with this. With respect to FGFR2 positivity, the rate of 25.6% in GC in our study is within the range reported for previous studies (2.5–61%) [10].

Among patients with CRC, we found that 16.2% were positive for HER2, which lies within the range of rates reported in previous studies (0.5–54%) [3]. The most recent studies tend to suggest that HER2 overexpression accounts for 1–6% of CRCs, with HER2-positivity rates of around 5% reported in *RAS* wild-type tumors [3, 11]. Consistent with this, we found that among our CRC patients, the proportion of HER2 IHC 3 + cases was greater in *RAS* wild-type cancers compared with *RAS*-mutated cancers. There have been few reports of FGFR2 expression levels in patients with CRC, but our rate of 2.9% is consistent with the 1.4% reported in a previous study [12]. Although it was previously reported that HER2 + CRC tumors are usually left-sided [13], HER2 status appeared to be the same for right-side and left-side primary cancer locations in our study.

Overall, we found no clear relationship between FGFR2 and HER2 status in either GC or CRC patients. Previous studies that focused specifically on amplifications found that HER2 and FGFR2 gene amplifications were usually mutually exclusive [14–16]. In our GC patients, neither FGFR2 nor HER2 expression status differed according to the primary cancer location; however, there were some differences between histological types. Previous reports have suggested that HER2 expression may vary between GC histological types, with higher rates reported for intestinal versus diffuse cancers [8, 9]. Although this study could not provide sufficient evidence to enable a clear conclusion to be reached, further investigation of the relationship between HER2 expression and GC histological type, with the molecular mechanism which defines the histological type, may provide insights to expand the indication for HER2 therapies. The limited number of FGFR2-positive CRC patients in our study meant that the relationship between FGFR2 and HER2 status in CRC could not be fully assessed with respect to differences between primary cancer locations and histological types. Despite this study not being able to reveal a relationship between FGFR2 and HER2 expression, it would be valuable to consider re-testing FGFR2 expression in tumor tissues of patients after HER2-targeted therapy to analyze the relationship between FGFR2 expression changes after HER2-targeted therapy and drug resistance, because studies have indicated the role of FGFR2 in HER2-targeted lapatinib resistance [17, 18].

Several studies have reported that gene amplification is generally present in CRC tumors that are strongly positive for HER2 overexpression on IHC [11, 19, 20]. Consistent with this, we found that CRC samples which were strongly positive for HER2 on IHC were also FISH positive.

More than 100 FGFR2- and/or HER2-positive patients were found in the current study, who were therefore potentially suitable for inclusion in clinical trials of targeted agents. Seven of these patients were successfully enrolled in other clinical trials, including DS1123-A-J101 (NCT02690337), DS8201-A-J101 (NCT02564900), and DS8201-A-J203 (NCT03384940). This suggests that the screening performed in the current study may be useful for identifying patients potentially eligible for clinical trials. This could be especially relevant for early phase trials targeting rare populations, such as patients with low biomarker prevalence. Moreover, this screening is a universal screening with IHC including pathological review by one pathologist to achieve standardized evaluation, with a cost of about US $1700 per patient, which is almost comparable to that of next-generation sequencing (NGS). Therefore, this screening method may be useful for efficient acceleration of studies which require an eligibility check with IHC.

We used a central laboratory to perform IHC and FISH for the current study. IHC of GC tissue samples was also performed at some local laboratories as part of routine clinical practice. This provided an opportunity to compare the results obtained in these different laboratory settings. We found that the results of HER2 IHC testing in GC matched between central and local laboratories in approximately 90% of cases (allowing for 1 level difference of IHC score). However, the rate of completely matched cases was only around 60%. The rate of matching was independent of whether or not the same sample was used for central and local testing (which could be assumed based on whether or not there was a difference in the sample collection dates) and was also independent of the type of IHC kit used. Overall, this suggests that improvements to, or standardization of, HER2 IHC laboratory methods may be desirable in the future.

In conclusion, this study determined the prevalence of FGFR2 and HER2 in Japanese patients with GC and CRC, and the values were concordant with previous reported prevalence rates. No clear relationship was found between FGFR2 and HER2 status in either the GC or CRC populations. FGFR2 and HER2 status did not differ according to the primary cancer location in GC, but there were some differences between GC histological types. These relationships could not be assessed properly in CRC, due to the limited number of FGFR2-positive patients. The screening performed in this study could be useful for identifying eligible patients for clinical trials of agents targeting these proteins.

## Supplementary Information

Below is the link to the electronic supplementary material.

Supplementary file1 (PDF 297 KB)

## Data Availability

There were no agreements from the participants for their data, so data sharing is not available.
