# Supplementary material for: Prospective analysis of the expression status of FGFR2 and HER2 in colorectal and gastric cancer populations: DS-Screen Study
Source: Int J Colorectal Dis. 2022 May 19;37(6):1393–402. doi: 10.1007/s00384-022-04162-2 (PMC9167213; doi:10.1007/s00384-022-04162-2)
Supplement: Supplementary file 1 — Supplementary file1 (PDF 297 KB) [file 384_2022_4162_MOESM1_ESM.pdf]

Online Resource – IHC score and positivity criteria

Prospective analysis of the expression status of FGFR2 and HER2 in colorectal and gastric cancer populations: DS-Screen Study

Hisateru Yasui, Atsushi Takeno, Hiroki Hara, Hiroshi Imamura, Hiroki Akamatsu, Kazumasa Fujitani, Minoru Nakane, Chihiro Nakayama Kondoh, Seigo Yukisawa, Junichiro Nasu, Yoshinori Miyata, Akitaka Makiyama, Hiroyasu Ishida, Norimasa Yoshida, Eiji Matsumura, Masato Ishigami, Masahiro Sugihara, Atsushi Ochiai, Toshihiko Doi

International Journal of Colorectal Disease

Corresponding Author: Toshihiko Doi, National Cancer Center Hospital East, Chiba, Japan

FGFR2, Gastric cancer/Colorectal cancer

IHC Score	Positivity	Criteria
3+	Positive	≥5% positive stained tumor cells with a strong membranous reactivity
2+		≥5% positive stained tumor cells with a weak to moderate membranous reactivity
1+		≥5% positive stained tumor cells with a weak membranous reactivity
0	Negative	No membranous reactivity or membranous reactivity in <5% of tumor cells

HER2, Gastric cancer, Surgical specimen

IHC Score	Positivity	Criteria
3+	Positive	Strong, complete basolateral or lateral membranous reactivity in ≥10% of tumor cells
2+		Weak to moderate, complete basolateral or lateral membranous reactivity in ≥10% of tumor cells
1+	Negative	Faint/barely perceptible membranous reactivity in ≥10% of tumor cells; cells reactive only in part of their membrane
0		No reactivity or membranous reactivity in <10% of tumor cells

HER2, Gastric cancer, Biopsy specimen

IHC Score	Positivity	Criteria
3+	Positive	Tumor cell cluster* with strong, complete basolateral or lateral membranous activity irrespective of percentage of tumor cells stained
2+		Tumor cell cluster with weak to moderate, complete basolateral or lateral membranous activity irrespective of percentage of tumor cells stained
1+	Negative	Tumor cell cluster with faint or barely membranous activity irrespective of percentage of tumor cells stained
0		No reactivity or membranous reactivity in any tumor cells

*"Tumor cell cluster" is defined as a cluster of 5 or more tumor cells

HER2, Colorectal cancer

IHC Score*		Positivity	Criteria			N (%)
			Intensity	Cellularity	Location	
3+	9	Positive	Intense	≥50%	Membrane Circumferential, basolateral or lateral	12 (3.1)
	8			≥10%, <50%		4 (1.0)
0	7	Negative		<10%		
2+	6	Positive	Moderate	≥50%		8 (2.1)
	5			≥10%, <50%		38 (9.9)
0	4	Negative		<10%		
1+	3		Faint	≥10%		94 (24.5)
0	2			<10%		
0	1		No staining	—		228** (59.4)

* For colorectal cancer, two HER2 IHC scoring criteria (0 to 3+, 1 to 9) were defined.

**IHC Score 1 includes score 2, 4, and 7.
